# *FGD3* as a prognostic and immunological biomarker: a pan-cancer analysis of its role in tumor progression and the immune landscape

**DOI:** 10.1186/s12935-026-04360-w

**Published:** 2026-06-10

**Authors:** Jing Zhu, Chun Yu, Xin Xu, Xin Zuo, Jiaqi Li, Rui Wang, Xinchao Li, Aijun Sun, Shiyan Wang, Chao Jiang

**Affiliations:** 1 Department of Oncology, The Affiliated Huaian No.1 People’s Hospital of Nanjing Medical University, Huai’an, 223300 China; 2 General Surgery Department, Lianshui People’s Hospital Affiliated to kangda college of Nanjing Medical University, Huai’an, Jiangsu China; 3 Department of Thyroid and Breast Oncological Surgery, Huai’an Second People’s Hospital, The Affiliated Huaian Hospital of Xuzhou Medical University, Huai’an, Jiangsu China; 4 Faculty of Life Science and Food Engineering, Huaiyin Institute of Technology, Huai’an, Jiangsu China

**Keywords:** *FGD3*, Pan-cancer, Tumor immunity, Prognosis, Immunotherapy drug susceptibility

## Abstract

**Background:**

*FGD3*, a member of the FGD family, regulates GTPase activity and cellular morphology, thereby influencing tumor proliferation and migration. However, its role across diverse cancer types remains insufficiently understood.

**Methods:**

Using TCGA and GTEx databases, *FGD3* expression was compared between tumor and normal tissues to assess its relevance to cancer. Single-cell transcriptomic data from TISCH were used to characterize *FGD3* expression across immune cell subsets. Univariate Cox regression was performed to evaluate associations between *FGD3* expression and patient survival. TIMER2.0 Spearman correlation analysis examined its relationship with immune cell infiltration. Gene set enrichment analysis (GSEA) and gene set variation analysis (GSVA) revealed *FGD3*’s association with immune and metabolic pathways. Bioinformatics and molecular docking predicted interactions with anticancer drugs. Experimental validation demonstrated that *FGD3* knockdown suppresses cancer cell migration and proliferation, indicating an anti-tumor role in breast cancer.

**Results:**

*FGD3* expression varies across tumors, with differing prognostic implications. In most malignancies, expression inversely correlates with copy number variation (CNV) and methylation and associates with immunotherapy biomarkers and responses. ESTIMATE and immune infiltration analyses suggest a role in immunosuppression and tumor immunity. Experimental downregulation of *FGD3* significantly reduces cell proliferation and migration, highlighting its therapeutic potential.

**Conclusion:**

*FGD3* plays an important role in tumorigenesis and progression and may serve as a promising biomarker for cancer prognosis. Its expression is inversely associated with tumor metastasis. Experimental findings further suggest that *FGD3* may function as a migration-suppressive factor in breast cancer. Collectively, these results provide new insights into the biological role of *FGD3* and suggest its potential as a therapeutic target.

**Supplementary Information:**

The online version contains supplementary material available at https://doi.org/10.1186/s12935-026-04360-w.

## Introduction

Cancer remains a major global health challenge, with increasing incidence and mortality imposing substantial burdens on healthcare systems worldwide [1]. Its development is driven by complex genetic and epigenetic alterations, highlighting the urgent need to identify novel diagnostic biomarkers and therapeutic targets. In this context, comprehensive pan-cancer analyses have emerged as a powerful strategy to systematically investigate transcriptomic landscapes and their clinical relevance [2, 3]. Large-scale initiatives such as TCGA Pan-Cancer Project have integrated multi-omics data to characterize both shared and tumor-specific molecular features, thereby providing a foundation for biomarker discovery and molecular classification of cancers [2, 4]. In addition, whole-genome analyses offer valuable insights into the molecular mechanisms underlying tumorigenesis [5].

Recent advances in high-throughput sequencing technologies and bioinformatics have transformed cancer research by enabling comprehensive characterization of the molecular features of tumors. These developments have accelerated the transition from conventional histopathological classification to molecularly informed diagnosis, thereby improving the precision of cancer classification and the development of targeted therapies [6]. In particular, large-scale cancer genomics projects such as TCGA have generated extensive genomic and clinical datasets, providing valuable resources for investigating the molecular basis of cancer [7].

The integration of multi-omics datasets has further enabled systematic pan-cancer analyses of gene expression patterns, clinical outcomes, and dysregulated signalling pathways across malignancies. These approaches not only deepen our understanding of tumor biology but also support the identification of novel biomarkers and therapeutic targets, thereby advancing the implementation of precision medicine in oncology.

The FGD gene family comprises six members (*FGD1*-*FGD6*) belonging to the FYVE domain-containing protein subfamily. These evolutionarily conserved proteins are characterized by multiple functional domains, including a FYVE domain, a Dbl homology (DH) domain, and two pleckstrin homology (PH) domains. Through interactions with small GTPases, members of the FGD family regulate diverse cellular processes, particularly actin cytoskeleton organization and cell morphology [8].

Individual FGD proteins exhibit distinct biological functions. For instance, *FGD1* has been implicated in tumor progression and has been reported to be abnormally upregulated in osteosarcoma, where it is associated with poor prognosis. Mechanistically, *FGD1* may activate the PI3K/AKT signaling pathway through interactions with PTEN [9]. *FGD2* functions as a guanine nucleotide exchange factor that activates CDC42 and is primarily expressed in antigen-presenting cells, including B cells, macrophages, and dendritic cells [10, 11].

Emerging evidence suggests that *FGD3* may act as a suppressor of cell migration and is associated with favorable prognosis in breast cancer patients [12, 13]. In addition, recent studies have identified a potential *FGD3*/HSF4/p65 signaling axis in pancreatic cancer [14]. High expression levels of *FGD2* and *FGD3* have also been linked to improved overall survival in head and neck squamous cell carcinoma [15]. Furthermore, *FGD3* has been reported as a potential downstream target of PPARgamma, implicating its involvement in thyroid cancer development [16].

Other members of the FGD family are also implicated in tumor biology. For example, *FGD5* has been shown to regulate VEGF-mediated angiogenesis in endothelial cells, while its antisense RNA, *FGD5*-AS1, plays a role in tumor progression through multiple mechanisms, including regulation of cytokine expression, microRNA sponging, and mitochondrial function [17–19]. Dysregulation of *FGD5*-AS1 has been associated with poor prognosis in several cancers, including pancreatic cancer, gastric cancer, and melanoma [20, 21].

Collectively, these findings indicate that the FGD gene family plays a critical role in tumor biology. However, the pan-cancer role of *FGD3*, particularly in relation to the tumor immune microenvironment, remains insufficiently explored.

*FGD3*, a member of the FGD family, encodes a guanine nucleotide exchange factor that regulates small Rho GTPases, thereby contributing to cytoskeletal organization, cell morphology, and migratory behaviour [22–24]. Through its role in actin cytoskeleton remodelling, *FGD3* has been implicated in the regulation of tumor cell motility, a process closely associated with cancer invasion and metastasis. Previous studies have demonstrated that elevated *FGD3* expression is associated with improved survival outcomes in breast cancer, whereas reduced expression correlates with increased recurrence risk, lymph node metastasis, and poor prognosis [24, 25]. These findings suggest that *FGD3* may function as an important regulator of tumor progression and may have potential clinical value as a prognostic biomarker.

In addition to its role in tumor cell migration, emerging evidence indicates that *FGD3* may be involved in broader tumor-associated biological processes, including immune regulation and therapeutic response. However, despite these observations, the expression characteristics, prognostic significance, and immunological relevance of *FGD3* across different tumor types have not been comprehensively evaluated. In particular, its relationships with genomic instability, tumor immune infiltration, and immunotherapy responsiveness remain poorly understood.

To address these gaps, a systematic pan-cancer analysis of *FGD3* was conducted using integrated datasets from TCGA, GTEx, the Human Protein Atlas (HPA), the Cancer Cell Line Encyclopedia (CCLE), and TISCH. The expression landscape of *FGD3* was comprehensively characterized, and its prognostic significance was evaluated across 33 cancer types. In addition, associations with genomic alterations, immune infiltration, and immunotherapy-related biomarkers were systematically investigated. The potential therapeutic relevance of *FGD3* was further assessed through drug sensitivity profiling, molecular docking analyses, and experimental validation in cancer cell models.

This study provides a comprehensive overview of the clinical and biological significance of *FGD3* across human cancers, highlighting its potential as a prognostic biomarker and therapeutic target, and offering new insights into its role in tumor progression and tumor-immune interactions.

## Methods

This study was approved by the Ethics Committee of the research institution (Approval No. HYIT-EC-2024-012).

### Public data and analytical methods

The Open Targets platform (URL: https://platform.opentargets.org/) was used to identify diseases and phenotypes associated with *FGD3*. Protein subcellular localization was obtained from the HPA (URL: https://www.proteinatlas.org/). RNA-seq data in transcripts per million (TPM) format were retrieved from the UCSC Xena database. Gene expression profiles of *FGD3* across 33 cancer types and corresponding normal tissues were analyzed. To ensure cross-dataset comparability, all data were uniformly processed using the Toil pipeline. Differential expression analysis between tumor and adjacent normal tissues was performed using the Gene_DE module in TIMER2.0 [26]. Expression data from cancer cell lines were obtained from the CCLE (URL: https://portals.broadinstitute.org/ccle/), covering 29 tissue types. Subsequent analyses were based on normalized TPM expression values and corresponding clinical annotations derived from TCGA Pan-Cancer cohort, downloaded from the Genomic Data Commons (GDC) portal [27]. Protein-protein interaction (PPI) networks were constructed using the GeneMANIA platform (URL: http://www.genemania.org/) to predict functional associations of *FGD3*, including physical interactions, co-expression, co-localization, genetic interactions, pathways, and shared protein domains [28]. In addition, two independent immunotherapy cohorts were included for validation analysis. The IMvigor210 cohort (*n* = 298), comprising urothelial carcinoma patients treated with anti-PD-L1 therapy (atezolizumab), was obtained from http://research-pub.gene.com/IMvigor210CoreBiologies/packageVersions/. The GSE91061 cohort, including 51 melanoma patients treated with anti-PD-1 therapy (nivolumab), was retrieved from the Gene Expression Omnibus (GEO, https://www.ncbi.nlm.nih.gov/geo/). Cancer type abbreviations are listed in Supplementary Table S1.

### Single-cell analysis and spatially resolved transcriptomics

Single-cell transcriptomic analysis was performed using the TISCH web server (URL: http://tisch1.comp-genomics.org/) [29], with parameters set to *FGD3* (gene), major lineage (cell type annotation), and all available cancer types. The expression distribution of *FGD3* across different cell populations was visualized using heatmaps. To further investigate the spatial distribution characteristics of *FGD3*, spatial transcriptomic analysis was conducted using the SpatialDB platform (URL: https://www.spatialomics.org/SpatialDB/) [30]. Specifically, *FGD3* expression patterns were examined in breast cancer tissues and analyzed for co-localization with canonical immune markers (CD8A and CD8B), representing cytotoxic T lymphocytes. In addition, the spatial association between *FGD3* and endothelial cell markers was assessed in colorectal cancer samples.

### Survival and prognostic analysis

The association between *FGD3* expression and patient survival outcomes, including overall survival (OS), disease-specific survival (DSS), disease-free interval (DFI), and progression-free survival (PFS), was evaluated using univariate Cox proportional hazards regression analysis implemented through the PanCanSurvPlot web platform (URL: https://smuonco.shinyapps.io/PanCanSurvPlot/). Transcriptomic and clinical data were obtained from TCGA database. For each cancer type, univariate Cox regression analysis was performed independently. To control for multiple comparisons across cancer types, p-values were adjusted using the Benjamini–Hochberg false discovery rate (FDR) method. Gene expression data generated from the Illumina HiSeq platform were used for survival analysis. Patients were stratified into high- and low-expression groups based on an optimal cutoff value determined separately for each cancer type. The cutoff value was determined using an optimization algorithm that identifies the expression threshold yielding the most significant difference in survival outcomes between groups. The results were presented as hazard ratios (HRs) and 95% confidence intervals (95% CIs) using the R package forestplot.

### Cancer-related genomic alterations and *FGD3* mutation profile

The incidence of four types of genomic alterations was analyzed (mutations, amplifications, deep deletions, and multiple alterations) in tumors using the cancer type summary module of cBioPortal (URL: https://www.cbioportal.org/) [31]. CNV data and their associations with *FGD3* mRNA expression were further investigated using the Gene Set Cancer Analysis (GSCA) platform (URL: http://bioinfo.life.hust.edu.cn/GSCA) [32]. Gene-level CNV statuses were categorized into four discrete states: heterozygous amplification, homozygous amplification, heterozygous deletion, and homozygous deletion. Corresponding *FGD3* mRNA expression data from TCGA were log₂-transformed as log₂(TPM + 1) prior to analysis. Spearman’s rank correlation analysis was performed to evaluate the association between CNV status (treated as an ordinal variable) and *FGD3* expression across samples within each cancer type. In addition, differential expression among CNV categories was assessed to determine the impact of copy number alterations on transcriptional regulation.

DNA methylation profiles of *FGD3* were systematically analyzed using the GSCA platform. The analysis focused on CpG sites located within the promoter region of *FGD3*, including CpG islands and adjacent shore regions upstream of the transcription start site, which are known to play key roles in transcriptional regulation. Methylation levels were quantified as β values ranging from 0 to 1, while mRNA expression data were obtained from TCGA and transformed as log₂(TPM + 1). Spearman’s rank correlation analysis was performed to assess the relationship between DNA methylation levels and *FGD3* expression across cancer types. Multiple hypothesis testing was adjusted using the Benjamini-Hochberg FDR method. Patients were stratified into high- and low-methylation groups for survival analysis. Differences in OS, DSS, and PFS between groups were evaluated using the log-rank test. Tumor heterogeneity was assessed using FDR-adjusted t-tests. Collectively, these analyses were used to evaluate the prognostic relevance of *FGD3* methylation and its potential impact on therapeutic response.

### Immunotherapy prediction analysis

To evaluate the potential association between *FGD3* expression and immunotherapeutic efficacy, somatic mutation data were obtained from TCGA via the UCSC Xena database (URL: https://tcga.xenahubs.net) and analyzed using the R package “maftools”. Tumor mutational burden (TMB) and microsatellite instability (MSI) scores were calculated for each TCGA sample. The correlations between *FGD3* expression and these genomic indicators were assessed using Spearman’s rank correlation analysis. In addition, correlations between *FGD3* expression and immune-related scores, including immune score, stromal score, and ESTIMATE score, were systematically evaluated. To control for multiple testing across cancer types, p-values were adjusted using the FDR correction. Results were visualized using radar plots generated with the R package “ggradar”. Immunotherapy response was classified into four clinical categories: progressive disease (PD), stable disease (SD), partial response (PR), and complete response (CR). For survival and response stratification, the optimal cutoff value of *FGD3* expression was determined using the surv_cutpoint function from the R package survminer. This approach applies maximally selected rank statistics to evaluate all possible dichotomization thresholds and identifies the cutpoint that best separates survival outcomes. Based on the optimized cutoff, patients from two independent immunotherapy cohorts were stratified into high- and low-*FGD3* expression groups. Survival outcomes and treatment responses were subsequently analyzed within each subgroup. Furthermore, the expression levels of mismatch repair (MMR) genes, including MLH1, MSH2, MSH6, PMS2, and EPCAM, were examined across cancer types. Their associations with *FGD3* expression were assessed using correlation analysis and visualized as heatmaps constructed with the R packages “tidyverse” and “ggnewscale”.

### Impact of *FGD3* expression on immune assessment

The extent of immune and stromal cell infiltration in tumor tissues was quantified using the ESTIMATE algorithm [33], which generates immune, stromal, and ESTIMATE scores based on gene expression signatures. Transcriptomic data derived from the Illumina sequencing platform were processed in R using the “limma” and “estimate” packages to evaluate the associations between *FGD3* expression and tumor microenvironment scores. The level of immune cell infiltration across different cancer types was further characterized using TIMER 2.0, which enables systematic evaluation of tumor-infiltrating immune cell abundance. Correlations between *FGD3* expression and multiple immune cell subsets were analyzed accordingly. To explore the immunological relevance of *FGD3* at the gene level, a curated list of 150 immune-related genes was retrieved from the TISDB database, including major histocompatibility complex (MHC) molecules, immune checkpoint genes, chemokines, chemokine receptors, and immune activators [34]. Associations between *FGD3* expression and these immune-related genes were evaluated using the R packages “limma”, “pheatmap”, and “ggplot2”, and visualized through heatmaps and correlation plots.

### Biological significance analysis of *FGD3* expression

To elucidate the biological functions of *FGD3* in tumor initiation and progression, GSEA and GSVA were performed. The R package “clusterProfiler” along with other relevant analytical tools, was utilized to interrogate the C2 and C5 gene sets [35]. Comparisons of the normalized enrichment score (NES) and FDR were conducted between low and high *FGD3* expression groups across diverse cancer types. The samples were classified into high-expression and low-expression groups based on the median level of *FGD3* gene expression. The significance of enrichment was determined at FDR < 0.05.

### Correlation between *FGD3* expression and drug sensitivity

To explore the potential link between *FGD3* and drug response, we utilized the CellMiner database (URL: http://discover.nci.nih.gov/cellminer/), which is specifically designed for cancer researchers. This resource consolidates molecular and pharmacological data from the NCI-60 cancer cell lines and is widely used for cancer drug screening [36]. Processed RNA expression data (based on RNA-seq technology) and compound activity data were downloaded from CellMiner, followed by screening of FDA-approved drugs and drugs in clinical trial stages. Data preprocessing was performed using the R package limma, which removed columns with more than 80% missing values, while the remaining missing data were imputed using the R package Impute. The results were visualized using the ggplot2 and ggpubr packages, with statistical significance set at *p* < 0.05.

Furthermore, molecular docking simulations were conducted using Autodock4 software to assess the binding affinity of docetaxel acetate (PubChem CID: 148124; URL: https://pubchem.ncbi.nlm.nih.gov/compound/148124) with the *FGD3* protein, based on drugs selected from the CellMiner, CTRP, and GDSC databases. The structure of docetaxel acetate was obtained from the PubChem database (URL: https://pubchem.ncbi.nlm.nih.gov/) [37, 38], while the three-dimensional structure of the *FGD3* protein was predicted using AlphaFold (URL: https://alphafold.ebi.ac.uk/) [39]. The docking analysis based on the AlphaFold-predicted *FGD3* structure is theoretical and intended only to suggest potential binding sites for future experimental validation. Finally, the docking model was visualized using Pymol software.

### Cell culture and transfection

The human breast cancer cell line MCF-7 and the liver adenocarcinoma cell line SK-Hep-1 were obtained from the Cell Bank of the Chinese Academy of Sciences, Shanghai, China. These cells were cultured in DMEM medium supplemented with 10% fetal bovine serum (FBS) provided by Procell (GenePharma, Shanghai). The cell culture was maintained at 37 °C under 5% CO2, and mycoplasma contamination was regularly monitored using PCR.

siRNAs targeting human *FGD3* and their negative controls were acquired from GenePharma (GenePharma, Shanghai). Following the manufacturer’s instructions, these siRNAs were transfected into the cells using the transfection reagent lipo3000 (Invitrogen, California, USA). Detailed siRNA sequences are provided in Supplementary Table S2.

### Reverse Transcription Quantitative Polymerase Chain Reaction (RT-qPCR)

Total RNA was extracted using TRIzol reagent (Takara Bio, Kusatsu, Japan), and the RNA concentration and purity were assessed with the NanoDrop 2000 system (Thermo Scientific). Reverse transcription PCR (RT-PCR) was performed using PrimeScript™ RT Master Mix (Takara, RR036A), and real-time quantitative PCR (qPCR) was conducted with SYBR^®^ Premix Ex Taq™ II (Takara, RR820A). Gene expression levels were analyzed using the 2-∆∆Ct method, with ACTB as the internal reference. The primer sequences for RT-qPCR are provided in Supplementary Table S3.

### Cell viability assay

Cell viability was assessed using the CCK-8 kit (GK10001, GLPBIO, Montclair, California, USA). Cells were seeded in 96-well plates at a density of 2 × 10^3^ per well, and 10 µl CCK-8 was added to each well after 24–48 h of incubation. After 2 h of incubation, the absorbance at 450 nm was determined using a microplate reader.

### Colony formation

For the colony formation assay, the MCF-7 and SK-Hep-1 cell lines were subjected to separate experiments, with 1,000 cells per well seeded in 6-well plates. Each condition was performed in triplicate. Fresh culture medium was replenished every three days. After one week of culture, cells were fixed with methanol and stained with 0.5% crystal violet. Clones were photographed and quantitatively analysed using ImageJ software.

### Cell scratch wound-healing assay

In the scratch healing assay, cells transfected with the indicated siRNAs were inoculated into 6-well plates with 1 × 10^5^ cells per well, and the medium was supplemented with 2% foetal bovine serum to avoid the effect of cell proliferation. The pipette gun tip was used to score the cell monolayer. Photographs of the healing area of the scratches were taken using a Nikon Ti-E inverted microscope (Nikon Instruments, Florence, Italy), and images were acquired at 0 and 48 h after scratching. Using ImageJ software, the area of the scratch at each time point was calculated and the area at each time point was normalised to a specific area at the moment of T0.

### PBMC co-culture and cytokine profiling by ELISA

Human peripheral blood mononuclear cells (PBMCs) were purchased from Shanghai Zhongqiao Xinzhou Biotechnology Co., Ltd. After thawing, the PBMCs were resuspended in RPMI-1640 complete medium supplemented with 10% fetal bovine serum, and cell viability exceeded 90%. MCF-7/ SK-Hep-1 non-targeting control (NC) and two *FGD3*-knockdown cell lines (*FGD3*-si1 and *FGD3*-si2) were routinely cultured in DMEM-F12 complete medium. For the co-culture assay, 5 × 10^4^ MCF-7/ SK-Hep-1 cells were seeded into the upper chamber of a 24-well Transwell plate (polycarbonate membrane, 0.4 μm pore size), while 2 × 10^6^ PBMCs were added to the lower chamber in RPMI-1640 complete medium, resulting in an effector-to-target ratio of 40:1. Co-cultures were maintained for 48 h at 37 °C under 5% CO_2_. The supernatants were then collected, centrifuged to remove debris, and stored at -80 °C for analysis. Cytokine concentrations (IFN-γ, IL-2, IL-10, and TGF-β1) in the supernatants were measured using commercially available sandwich ELISA kits (R&D Systems) according to the manufacturer’s instructions. Briefly, after coating with capture antibody and blocking, standards and samples were added to the wells, followed by incubation with detection antibody, streptavidin-HRP, and TMB substrate. The reaction was stopped with 2 N H_2_SO_4_, and absorbance was measured at 450 nm. Cytokine concentrations (pg/mL) were determined by interpolation from the respective standard curves.

### Statistical analysis

The dataset was screened to remove missing values and duplicate entries, followed by a log_2_(TPM + 1) transformation on the TPM values. The Mann-Whitney U test (also known as the Wilcoxon rank-sum test) was used to evaluate the statistical significance of *FGD3* expression levels between normal and tumor tissues. For data from different tissue origins in the CCLE database, the Kruskal-Wallis test was applied to analyze *FGD3* expression. Depending on whether the samples were paired, paired t-tests or unpaired t-tests were used to compare the heterogeneity of *FGD3* expression levels between different groups or between tumor and normal tissues. For analyses involving multiple comparisons (including correlation, enrichment, immune infiltration, and drug sensitivity analyses), p-values were adjusted using the Benjamini-Hochberg FDR method. Adjusted q < 0.05 was considered statistically significant, unless otherwise specified. For single-cancer survival analyses, raw p-values are reported as only one gene was tested per model. All data analyses were conducted using R software (version 4.4.0; https://www.R-project.org).

## Results

### Differential expression of *FGD3* in PanCancer

**Fig. 1:**
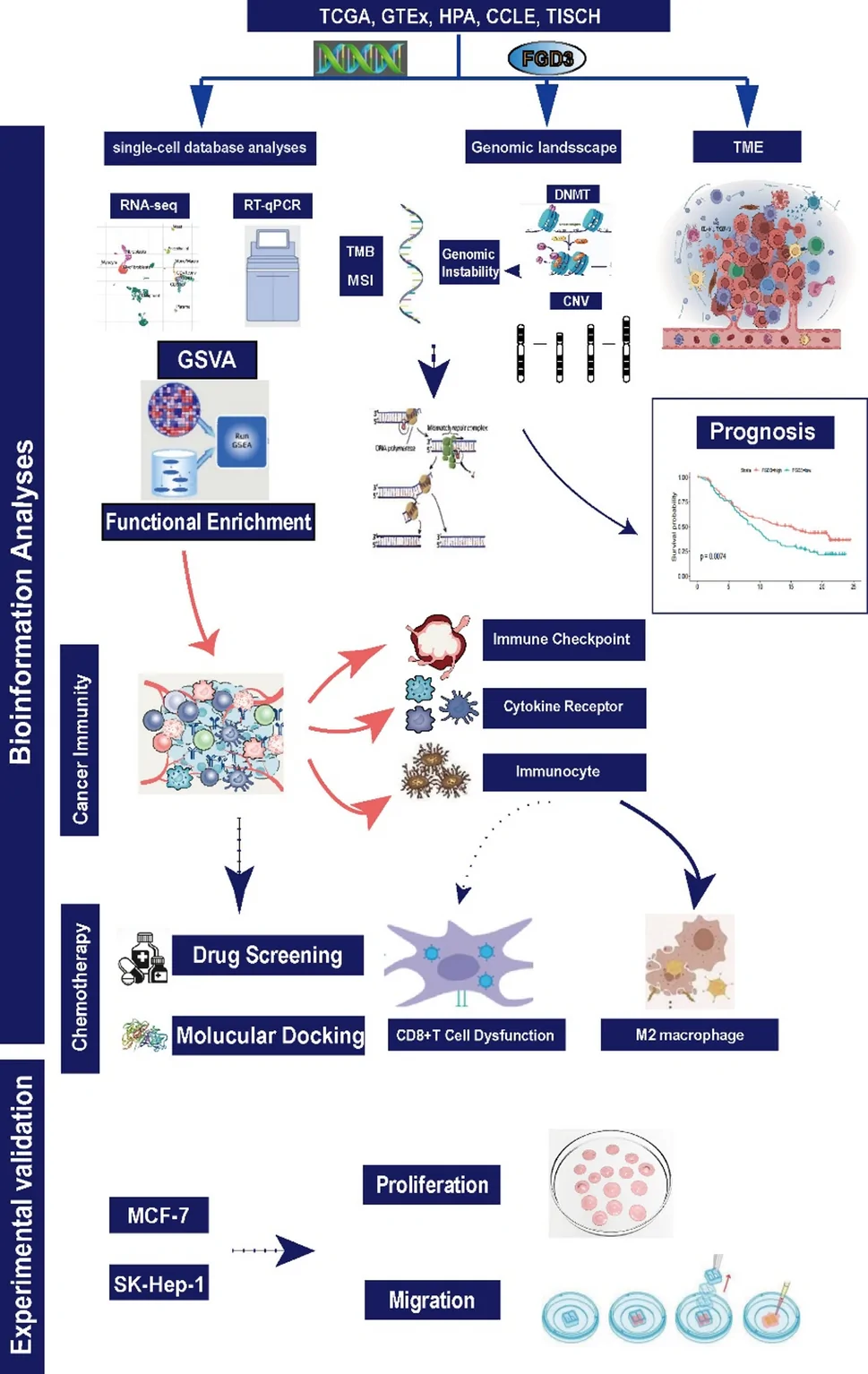
The study’s methodological framework delineates a comprehensive investigation of the target gene *FGD3* across cancers. Initially, *FGD3* expression was evaluated in a pan-cancer analysis, followed by an exploration of its associations with various cancer subtypes. The analysis compared *FGD3* expression across 33 malignant and non-malignant tissues, as well as distinct cellular environments. Subsequent investigations incorporated single-cell transcriptomic analyses and prognostic evaluations. A comprehensive examination of the genomic landscape was performed, with a focus on genomic instability. Data from the cBioPortal and GSCA databases were used to assess pan-cancer alterations, including CNVs and DNA methylation. Furthermore, the correlations between *FGD3* expression and TMB, MSI, and MMR status were analyzed. To elucidate the functional role of *FGD3* in carcinogenesis, functional enrichment analysis was conducted, along with evaluations of immune checkpoint pathways, cytokine-receptor interactions, and immune cell infiltration. Finally, the potential therapeutic implications of *FGD3* were assessed through predictions of chemotherapy response, drug sensitivity profiling, and experimental validation. Statistical significance was defined as an FDR-adjusted p-value < 0.05

**Fig. 2:**
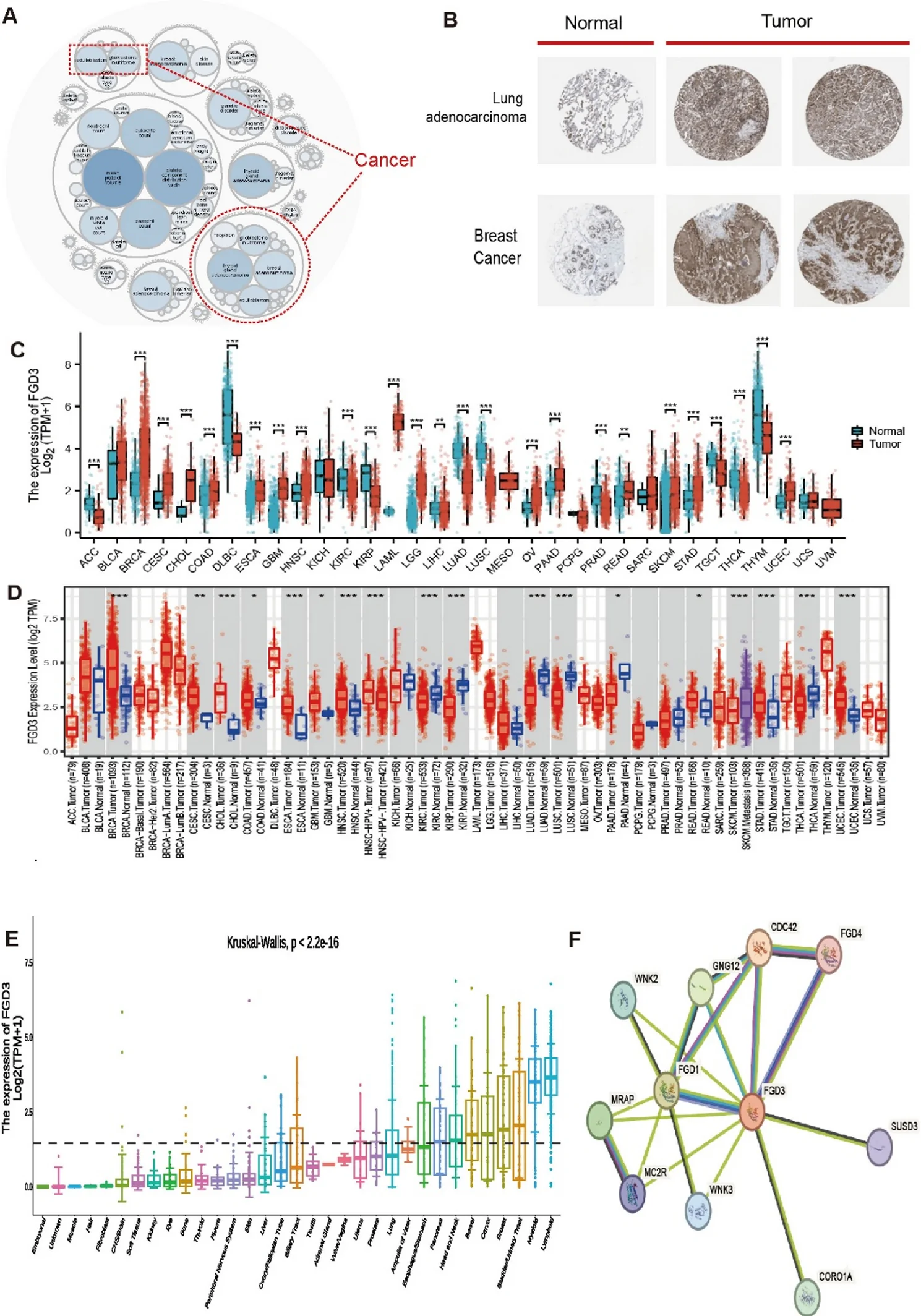
*FGD3* expression in human cancers. Diseases associated with *FGD3* identified using the Open Targets platform are shown in Fig. 2A, with *FGD3*-associated cancers highlighted by dashed red boxes. Protein expression of *FGD3* in normal (left) and tumor (right) tissues, including lymph nodes and breast tissue, was assessed by immunohistochemical staining, as shown in Fig. 2B. Differences in *FGD3* expression between tumor and normal tissues across 33 cancers were analyzed using combined TCGA and GTEx data, as shown in Fig. 2C. *FGD3* mRNA expression in various cancers based on TIMER2.0 is shown in Fig. 2D, with tumor tissues colored in red, normal tissues in blue, and SKCM metastatic tissues in purple. *FGD3* expression across 29 tissues based on CCLE data is shown in Fig. 2E. The PPI network of *FGD3* is shown in Fig. 2F, where different colored lines represent distinct prediction methods. Statistical significance was defined as an FDR-adjusted p-value < 0.05. Scale bar, 100 μm

Figure 1 illustrates the overall scope of the study. *FGD3* expression was systematically analyzed across multiple cancer types to characterize its pan-cancer transcriptional landscape. Disease association analysis using the Open Targets platform revealed that *FGD3* is linked to multiple malignancies, including glioblastoma multiforme, breast cancer, lung adenocarcinoma, and neuroblastoma (Fig. 2A). Immunohistochemical data from the Human Protein Atlas demonstrated higher *FGD3* protein abundance in tumor tissues compared with normal counterparts in both breast cancer and lung adenocarcinoma (Fig. 2B), suggesting its involvement in tumorigenesis.

Pan-cancer transcriptomic analysis integrating TCGA and GTEx datasets showed significant dysregulation of *FGD3* across diverse tumor types. *FGD3* was markedly upregulated in BLCA, BRCA, GESC, CHOL, COAD, ESCA, GBM, HNSC, LAML, LGG, PAAD, and UVM, whereas decreased expression was observed in ACC, DLBC, KIRC, LUAD, LUSC, PRAD, TGCT, and THYM (Fig. 2C). Independent validation using TCGA data confirmed these findings and further identified significant overexpression in ACC, BLCA, BRCA, CESC, CHOL, LGG, LIHC, LUAD, LUSC, and THYM (Fig. 2D), highlighting substantial inter-tumor heterogeneity. Analysis of normal tissue expression revealed that *FGD3* is highly expressed in soft tissues and is enriched in bone marrow-derived immune cells, including lymphocytes (Fig. 2E), suggesting a potential role in immune-related biological processes. On the basis of these expression characteristics, potential molecular mechanisms underlying *FGD3* function in tumorigenesis were further explored through protein-protein interaction analysis. *FGD3* was identified as a central hub within the interaction network, displaying close connectivity with *FGD1*, CDC42, *FGD4*, GNG12, WNK2, MRAP, MC2R and WNK3 (Fig. 2F). Collectively, these multi-layered analyses - encompassing disease association, protein-level validation, pan-cancer expression profiling and interaction network architecture - converge to indicate that *FGD3* may play a critical role in tumor development and provide a conceptual framework for subsequent investigation of its clinical and immunological significance.

### Expression of *FGD3* in different cells within the tumor microenvironment (TME) by using single-cell transcriptomic analysis

**Fig. 3:**
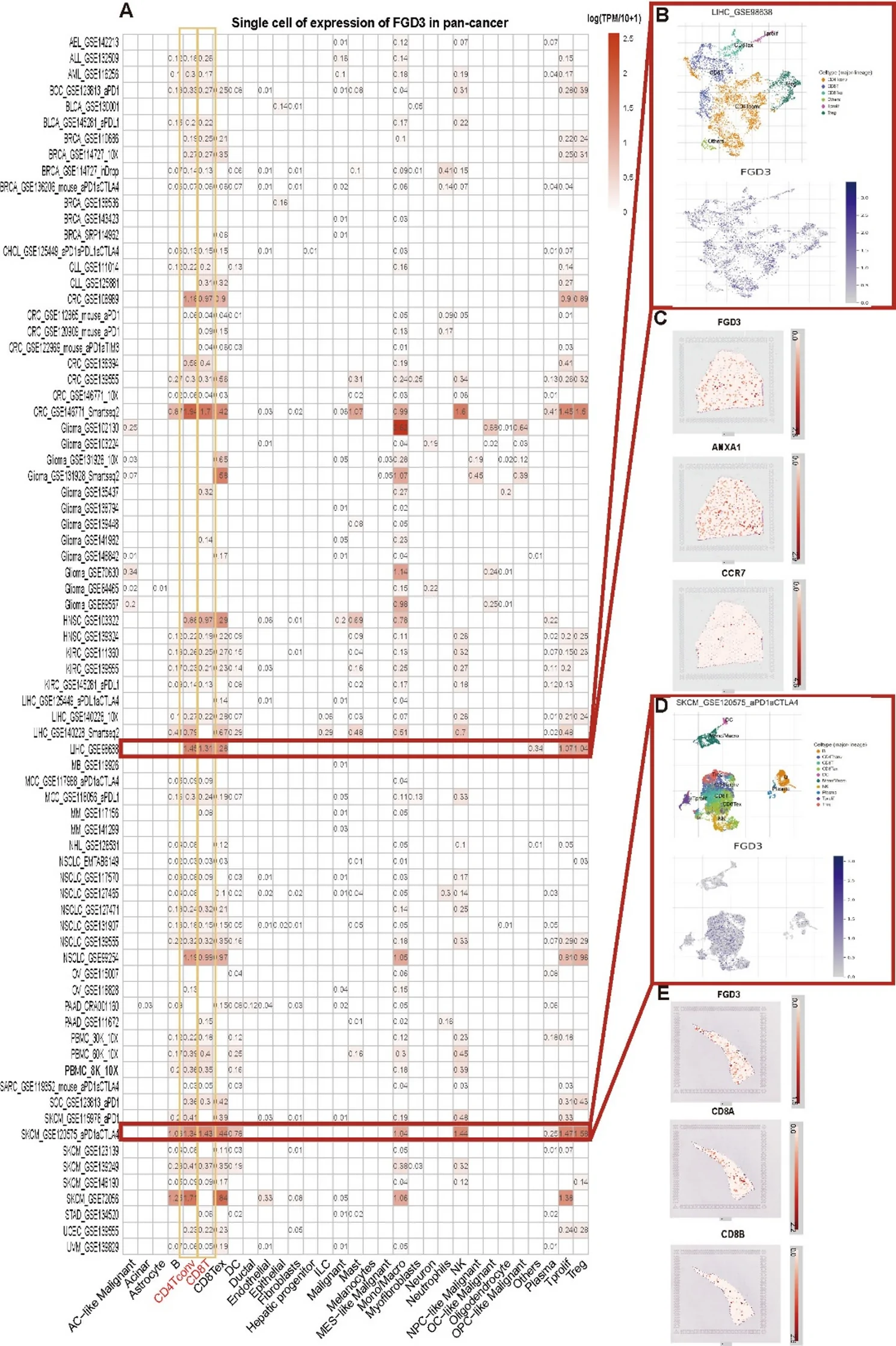
Distribution of cell types and spatial expression characteristics of *FGD3* expression. Aggregated *FGD3* expression across 33 cell types from 77 single-cell datasets is shown in Fig. 3A. The distribution of six cell types and their *FGD3* expression levels in the GSE110686 BRCA dataset is shown using a scatterplot in Fig. 3B. The distribution of 13 cell types and their *FGD3* expression levels in the GSE146771 CRC dataset is shown using a scatterplot in Fig. 3C. Spatial transcriptomic sections illustrating the spatial expression of ANXA1 and CCR7 are shown in Fig. 3D, with dot color indicating expression levels. Spatial transcriptomic sections illustrating the spatial expression of *FGD3*, CD8A, and CD8B are shown in Fig. 3E, with dot color indicating expression levels. Statistical significance was defined as an FDR-adjusted p-value < 0.05

To delineate the cellular origin of *FGD3* within the tumor microenvironment and assess its immunological relevance, a systematic single-cell transcriptomic analysis was performed across 76 cancer datasets. The TISCH-derived heatmap (Fig. 3A) illustrated the distribution of *FGD3* expression across 31 distinct cell populations, including immune, stromal and malignant compartments as well as other functional cell types. *FGD3* expression was largely restricted to immune cell subsets. In the LIHC-GSE98638 cohort, comprising 5,063 cells from 31 patients, *FGD3* was broadly detected in tumor-infiltrating immune cells, with particularly high enrichment in T lymphocytes (Fig. 3B). A highly consistent expression pattern was observed in the SKCM-GSE120575 dataset (16,291 cells from 31 patients), further supporting the conclusion that *FGD3* predominantly originates from immune cell populations within the tumor microenvironment. A more refined analysis of the LIHC microenvironment demonstrated significant *FGD3* expression in multiple immune cell subsets, including T cells, monocytes and macrophages (Fig. 3D), suggesting its involvement in immune cell-associated biological processes.

To validate these observations in a spatial context, spatial transcriptomic analysis was conducted in parallel with the assessment of canonical CD4⁺ T-cell markers (ANXA1 and CCR7) and CD8⁺ T-cell markers (CD8A and CD8B). The signal intensity in Fig. 3C and E corresponds to the expression levels of these marker genes. The spatial distribution of *FGD3* closely paralleled that of T-cell marker genes, providing orthogonal evidence that *FGD3* is predominantly localized to immune-infiltrated regions. Collectively, the concordant single-cell and spatial transcriptomic data indicate that *FGD3* is primarily derived from immune components of the tumor microenvironment, particularly T lymphocytes, thereby establishing a cellular foundation for subsequent analyses of its immune infiltration patterns and clinical significance.

### Prognostic value of *FGD3* expression levels in multiple cancer types

**Fig. 4:**
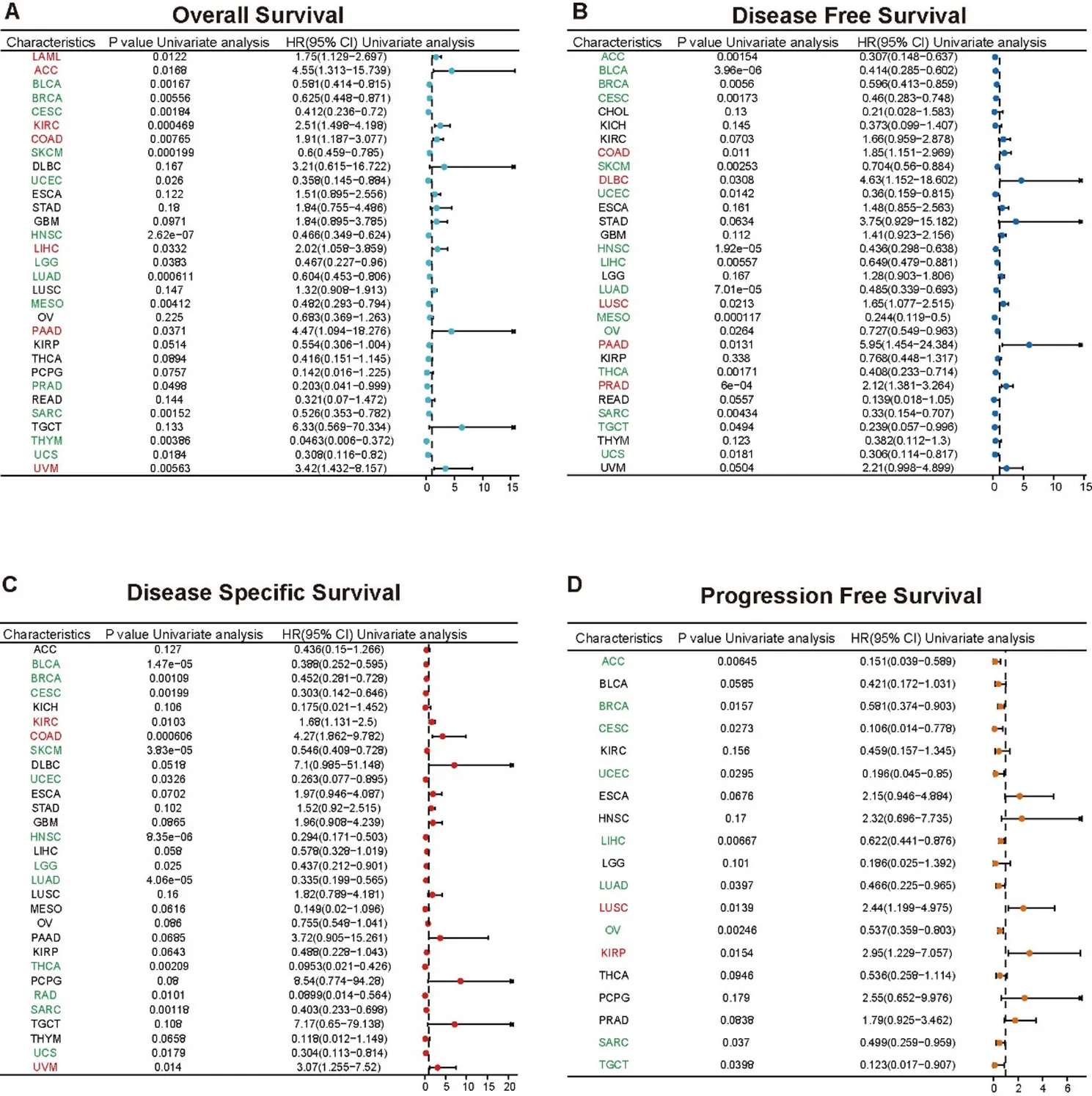
Univariate Cox regression analysis of the prognostic value of *FGD3* expression across cancers. Forest plots showing the association between *FGD3* expression and OS, DFS, DSS, and PFS are presented in Fig. 4A, B and C, and Fig. 4D, respectively. Risk factors with HR > 1 are indicated in red, whereas favorable factors with HR < 1 are indicated in green. All correlations were adjusted for multiple testing using the Benjamini–Hochberg method

Univariate Cox regression analysis based on TCGA datasets was performed to evaluate the clinical relevance of *FGD3* across tumor types and to systematically investigate the association between *FGD3* expression and patient prognosis. Survival analyses were conducted for OS, DSS, disease-free survival (DFS) and PFS. In general, *FGD3* expression showed significant prognostic relevance in multiple cancers, whereas no significant association was observed in BLCA, BRCA, CESC, COAD, UCEC, HNSC, LUAD, MESO, PRAD and THYM. In the OS analysis, elevated *FGD3* expression was associated with favourable prognosis in ACC, DLBC, STAD, GBM, LIHC, PAAD and TGCT, while it acted as a risk factor in BLCA, BRCA, CESC, COAD, UCEC, HNSC, LUAD, MESO, PRAD and THYM (Fig. 4A). In the DFS analysis, *FGD3* exhibited a protective effect in DLBC, STAD, PAAD and UVM and was also correlated with prolonged DFS in ACC, BLCA, BRCA, CESC, SKCM, UCEC, HNSC, LIHC, LUAD, MESO, OV, THCA, SARC, TGCT and UCS (Fig. 4B). These findings derived from the Cox proportional hazards model highlight the pronounced prognostic heterogeneity of *FGD3* across tumor types. To minimize the confounding effect of non-cancer-related mortality and to more accurately assess tumor-specific survival, DSS analysis was subsequently performed. *FGD3* functioned as a protective factor in COAD, DLBC, PAAD, PCPG, TGCT and UVM, whereas it was identified as a risk factor in BLCA, BRCA, CESC, SKCM, UCEC, HNSC, LGG, LUAD, THCA, READ, SARC and UCS (Fig. 4C). In the PFS analysis, *FGD3* was associated with adverse prognosis in LUSC and KIRP, but correlated with favourable outcomes in ACC, BRCA, CESC, UCEC, LIHC, LUAD, OV, SARC and TGCT (Fig. 4D).

The prognostic impact of *FGD3* exhibited a bidirectional and tumor-type-dependent pattern, suggesting that its functional role may be modulated by tumor-intrinsic molecular context and the heterogeneity of the tumor microenvironment. These observations provide a clinical rationale for subsequent investigations into the genomic regulatory mechanisms and immunological features associated with *FGD3*.

### Alterations in the *FGD3* gene are altered in pan-cancer and are associated with genomic instability and aberrations in carcinoma

**Fig. 5:**
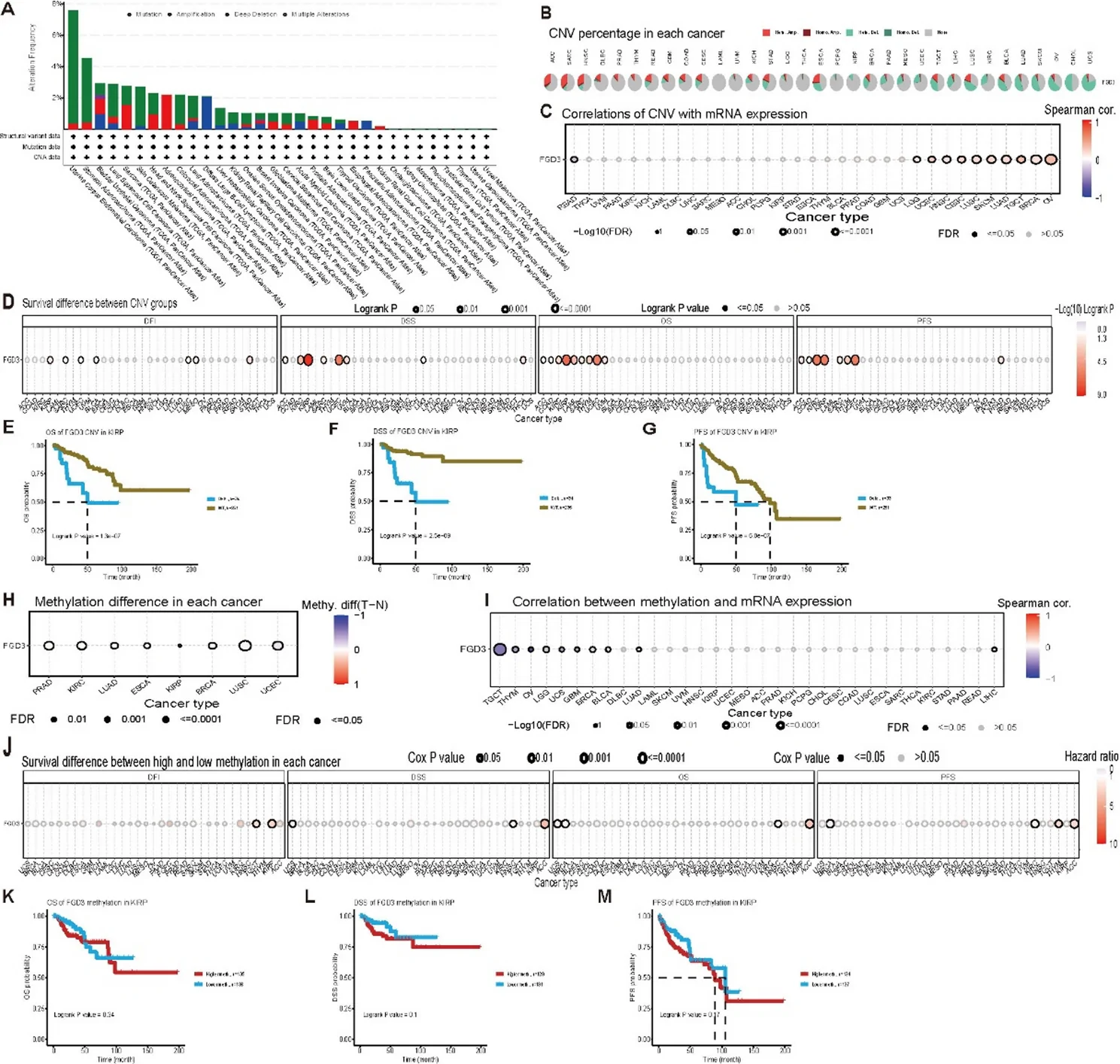
*FGD3* is associated with genomic instability in TCGA tumors. Genomic alterations of *FGD3*, including mutations, amplifications, deep deletions, and multiple alterations, across the TCGA pan-cancer cohort are shown in Fig. 5A. CNV of *FGD3* across various cancer types is summarized in Fig. 5B. The relationship between *FGD3* CNV and mRNA expression based on Spearman correlation analysis is shown in Fig. 5C. The correlation between *FGD3* CNV status and OS, DSS, PFS, and DFI is shown in Fig. 5D. Kaplan-Meier survival curves generated using the GSCA web tool for ACC patients are shown in Fig. 5E-G. Methylation differences in cancers with more than 10 tumor-normal sample pairs are shown in Fig. 5H, with only results meeting p-value ≤ 0.05 displayed. The correlation between *FGD3* methylation and mRNA expression based on Spearman correlation analysis is shown in Fig. 5I. The correlation between *FGD3* methylation status and OS, DSS, PFS, and DFI is shown in Fig. 5J. Kaplan-Meier survival curves showing survival outcomes of ACC patients stratified by high and low *FGD3* methylation status are presented in Fig. 5K-M. All correlations were adjusted for multiple testing using the Benjamini-Hochberg method

A systematic analysis of *FGD3* genomic alterations across diverse cancers was performed using the cBioPortal database to explore the molecular basis of its heterogeneous biological roles. The highest alteration frequency was observed in uterine endometrial carcinoma (> 6%), with mutations representing the predominant alteration type (Fig. 5A). Considerable variation in *FGD3* alteration frequency was evident across cancer types: CNV was particularly prevalent in ACC and SARC, whereas mutations dominated in most other malignancies, suggesting that distinct genomic perturbations of *FGD3* may contribute to oncogenesis through diverse mechanisms.

Further evaluation of CNV and DNA methylation status was conducted using the GSCA database. Pie charts illustrated the proportion of samples with heterozygous amplification, heterozygous deletion, or no CNV. Bubble plot analysis demonstrated significant correlations between CNV levels and *FGD3* mRNA expression in multiple cancers, including ACC, CHOL, PCPG, KIRP, STAD, ESCA, THYM, BLCA, PRAD, COAD, GBM, UCS, LGG, HNSC, CESC, LUSC, BRCA and OV. Compared with heterozygous deletions, amplification and no-CNV states were more prevalent, indicating a positive association between CNV and *FGD3* expression, with amplification corresponding to higher transcript levels (Fig. 5B, C).

The clinical significance of these genomic alterations was assessed by stratifying samples according to DFI, DSS, OS and PFS. Patients with high CNV exhibited reduced survival in KIRC, KIRP, UCEC, LGG, THCA and PRAD, with KIRP consistently displaying adverse outcomes across multiple survival metrics (Fig. 5D-G), implying that CNV-mediated modulation of *FGD3* expression may influence patient prognosis. DNA methylation, as a key epigenetic regulatory mechanism, was also examined. Compared with normal tissues, *FGD3* methylation levels were elevated in PRAD, KIRC, LUAD, ESCA, BRCA, UCEC and KIRP, whereas LUSC exhibited a decrease (Fig. 5H). Correlation analysis revealed a negative association between methylation and *FGD3* expression in UCS, in contrast to a positive correlation in LIHC (Fig. 5I), highlighting tumor-type-specific regulatory patterns. Stratification of tumors based on median methylation levels demonstrated that hypermethylation was associated with reduced overall survival in TGCT, KIRP, KIRC and ACC, and similarly indicated poor prognosis in UCS, BRCA and HNSC (Fig. 5J). Interestingly, in KIRP, high *FGD3* methylation was associated with lower mortality across several survival endpoints (Fig. 5K-M), suggesting context-dependent effects.

Collectively, CNV and DNA methylation appear to be critical upstream regulators of *FGD3*, jointly modulating its expression and potentially contributing to its heterogeneous prognostic effects across cancers.

### Co-expression of *FGD3* with immune-related genes

TMB and MSI are established determinants of sensitivity to immune checkpoint inhibitors, exerting a pronounced influence on patient prognosis and therapeutic response [40, 41]. TMB was quantified by enumerating somatic nonsynonymous mutations within the coding regions of tumor genomes [42], whereas MSI was assessed by calculating MMR deficiencies per million base pairs [43]. MMR plays a critical role in correcting DNA replication errors during cell division, and somatic mutations accumulate when MMR function is impaired.

**Fig. 6:**
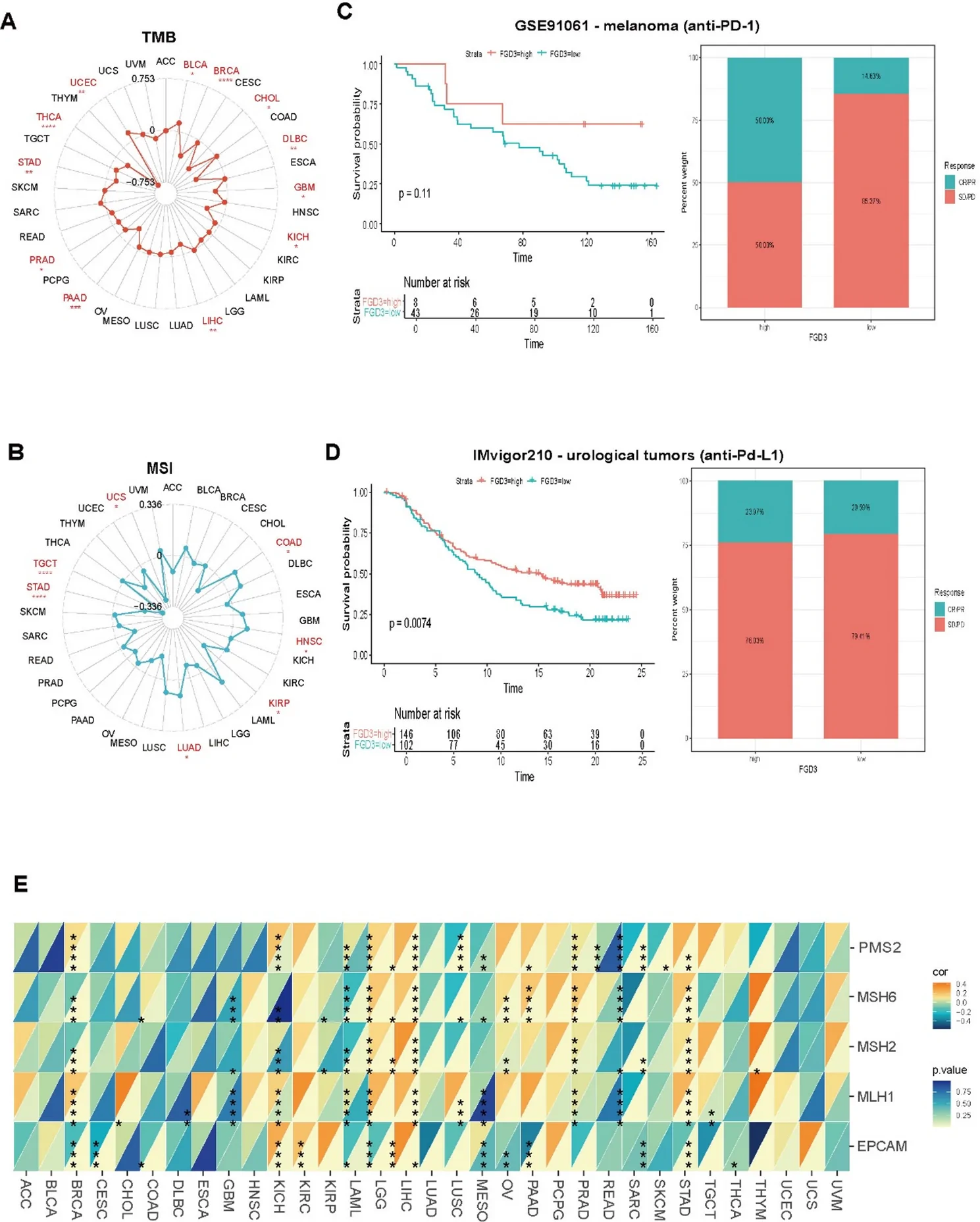
Association of *FGD3* expression with TMB, MSI, and MMR across cancer types. The relationship between *FGD3* expression and TMB is shown in Fig. 6A using radar plots, and MSI in Fig. 6B. Kaplan-Meier survival curves and proportions of immunotherapy response in low and high *FGD3* expression groups are shown for two independent cohorts. For the GSE91061 cohort (top), results are presented in Fig. 6C. For the IMvigor210 cohort (bottom), results are also presented in Fig. 6D. The correlation between *FGD3* expression and five MMR genes in a pan-cancer cohort is shown in Fig. 6E using a heatmap. Statistical significance is indicated as *p < 0.05, **p < 0.01, ***p < 0.001, and ****p < 0.0001. All correlations were adjusted for multiple testing using the Benjamini-Hochberg method

The association between *FGD3* expression and TMB across diverse cancer types was evaluated (Fig. 6A, B). Positive correlations were observed in BLCA, UCEC and SARC, whereas negative correlations were evident in THYM, THCA, STAD, BRCA, CHOL, DLBC, KICH and LIHC. Similarly, *FGD3* showed positive correlations with MSI in BLCA, COAD, DLBC, HNSC, LAML, LUAD, LUSC and UVM, but negative correlations in UCS, ACC, CHOL, KIRP, LGG, LIHC, MESO, READ, STAD and THYM. To explore the potential of *FGD3* as a predictive biomarker for immunotherapy response, two independent patient cohorts receiving anti-PD-1 or anti-PD-L1 therapy were analyzed. In a melanoma cohort, high *FGD3* expression was associated with improved survival following anti-PD-1 treatment, although the objective response rate remained moderate (50.0%) (Fig. 6C). Similarly, in the IMvigor210 cohort, elevated *FGD3* expression was associated with enhanced sensitivity to immunotherapy (Fig. 6D). The potential relationship between *FGD3* and genomic stability was further examined through its correlation with key MMR genes, including MLH1, MSH2, MSH6, PMS2 and EPCAM. Across pan-cancer datasets, *FGD3* expression was positively correlated with MMR gene expression, most prominently in BRCA, KIRC, LGG, OV, PAAD, PCPG, STAD and TGCT, with the strongest associations in KICH, LIHC and THYM (Fig. 6E).

These findings indicate that *FGD3* is associated with TMB, MSI and MMR gene expression, and may have predictive relevance for immunotherapy response, suggesting a potential link to tumor immunity through genomic stability.

### Association of *FGD3* expression with immune-related factors

**Fig. 7:**
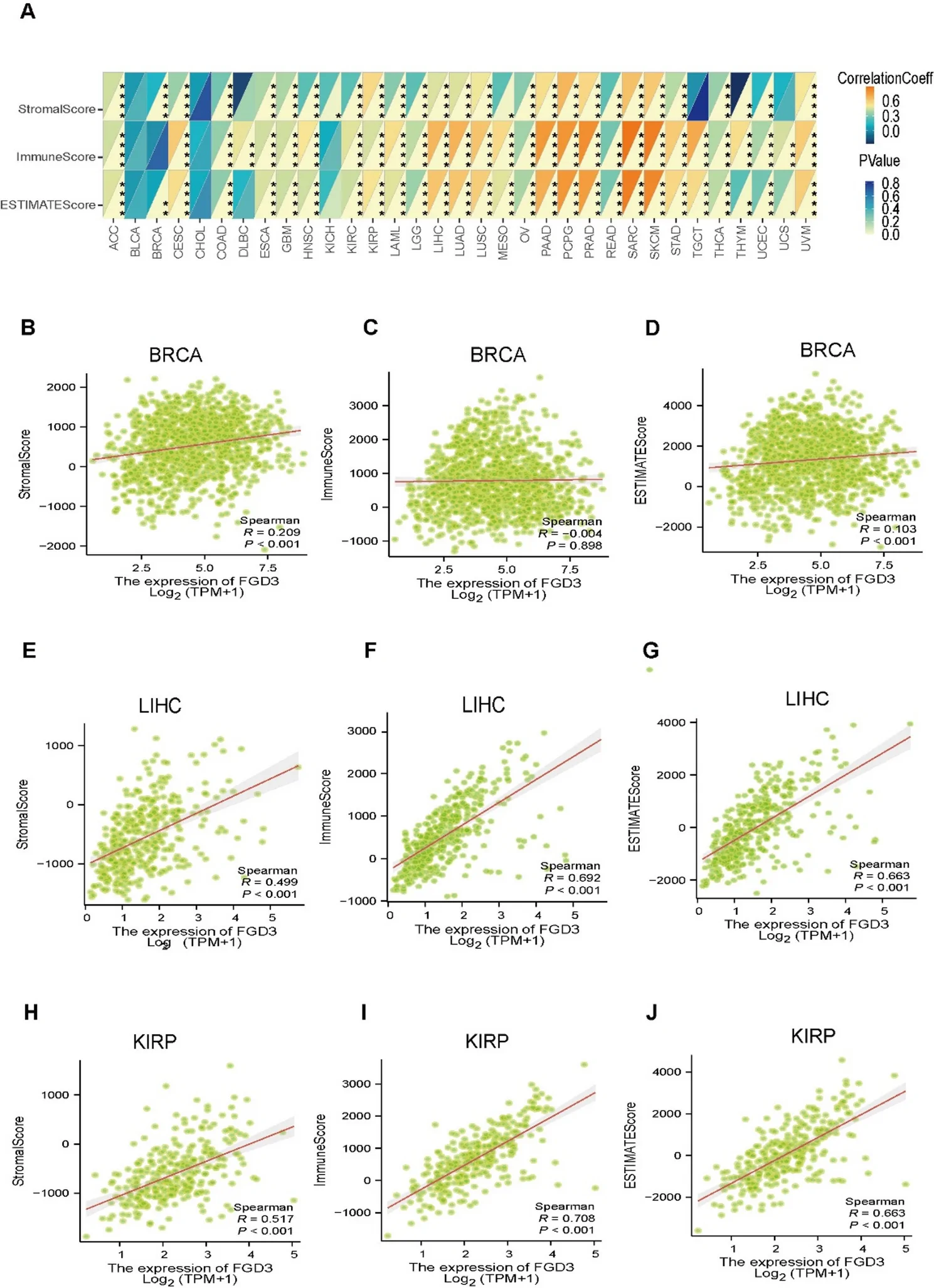
Analysis of *FGD3* expression and immune characteristics. The relationships between *FGD3* expression and ImmuneScore, StromalScore, and EstimateScore are shown in Fig. 7A based on Spearman correlation analysis, with correlation coefficients displayed in the upper-left triangle and p-values in the lower-right triangle. Significance levels are indicated as *p < 0.05, **p < 0.01, ***p < 0.001, and ****p < 0.0001. The relationships between *FGD3* expression and these three scores in ACC, BRCA, and UCEC are shown in Fig. 7B-D and E-G, and Fig. 7H-J, respectively. All correlations were adjusted for multiple testing using FDR, with statistical significance defined as p-value < 0.05

TME comprises a complex ecosystem of stromal cells, fibroblasts, endothelial cells, and both innate and adaptive immune populations, which collectively influence tumor progression and therapeutic response. Given the prior single-cell analyses indicating that *FGD3* is predominantly derived from immune cells, the relationship between *FGD3* expression and TME composition was further investigated. A comprehensive assessment of StromalScore, ImmuneScore, and ESTIMATE scores across diverse cancer types was performed to systematically evaluate correlations between *FGD3* expression and the various TME components (Fig. 7A). *FGD3* expression was found to be significantly negatively correlated with StromalScore, ImmuneScore, and ESTIMATE scores in multiple tumor types, with the strongest associations observed in cancers exhibiting the most pronounced variation in these metrics.

Detailed analyses in BRCA, LIHC, and KIRP further corroborated these observations (Fig. 7B-J), demonstrating consistent negative correlations between *FGD3* expression and overall TME scores. These findings suggest that *FGD3* may contribute to tumor microenvironment remodeling beyond reflecting immune cell abundance, supporting further investigation into its role in immune infiltration and function.

### Correlation between *FGD3* expression and immune permeable/immunomodulatory genes

**Fig. 8:**
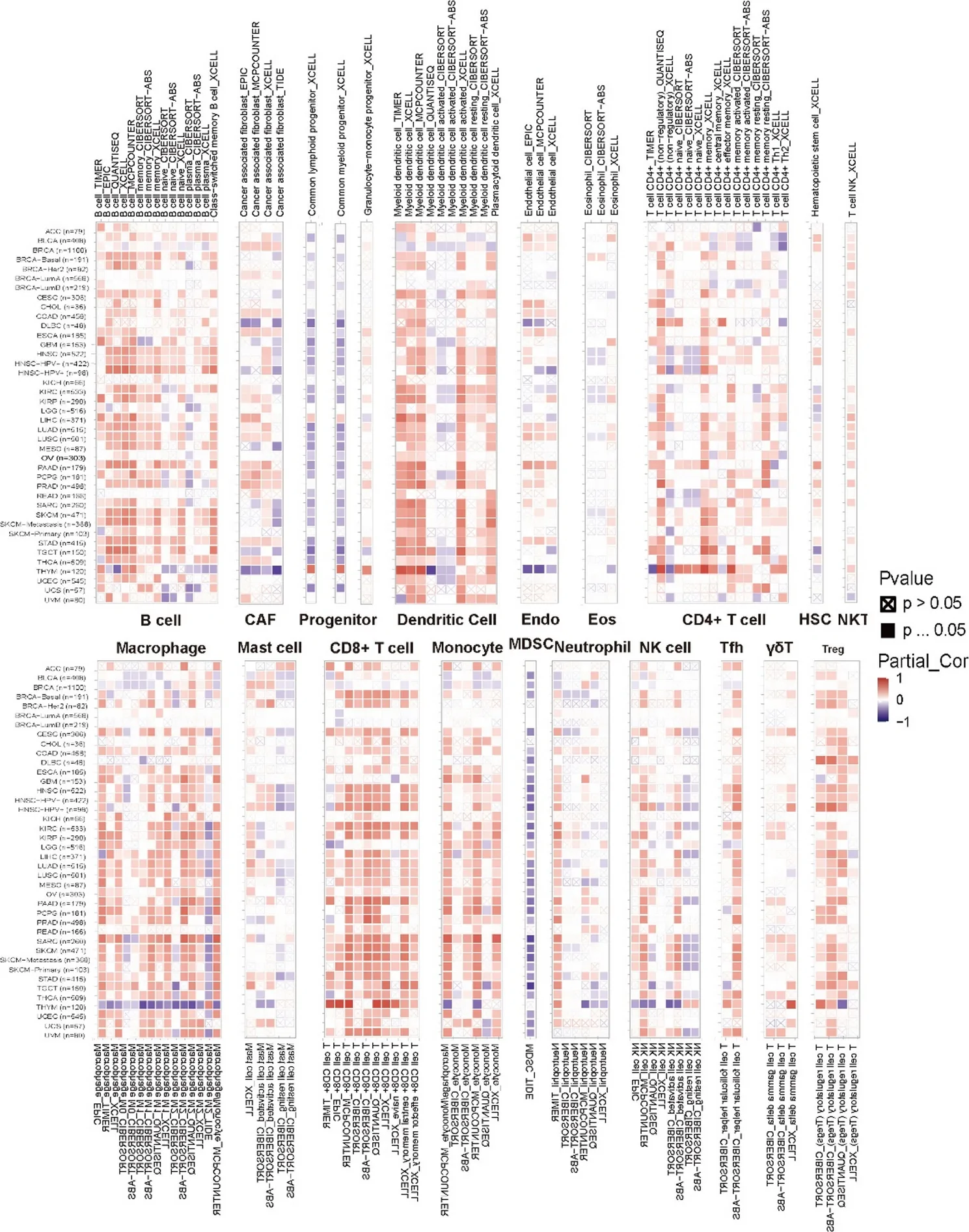
*FGD3* expression in relation to immune cell infiltration. Correlations between *FGD3* expression and CD4 + T cells, CAFs, precursor cells, endothelial cells (Endo), eosinophils (Eos), hematopoietic stem cells (HSC), Tfh cells, γδ T cells, NKT cells, Tregs, B cells, neutrophils, monocytes, macrophages, dendritic cells, NK cells, mast cells, and CD8 + T cells across multiple malignancies were analyzed using data from the TIMER 2.0 database and TCGA. Positive correlations are indicated in red and negative correlations in blue. Statistical significance corresponds to FDR-adjusted p-value < 0.05 across cancer types

To investigate the potential role of *FGD3* in tumor immune regulation, the association between its expression and the infiltration levels of diverse immune cell types was systematically examined. *FGD3* expression exhibited significant correlations with multiple immune cell types, including B cells, CAFs, progenitors, dendritic cells, endothelial cells, eosinophils, CD4 + T cells, HSCs, NKT cells, macrophages, mast cells, CD8 + T cells, monocytes, MDSCs, neutrophils, NK cells, T follicular helper cells (Tfh), γδ T cells, and regulatory T cells (Tregs) (Fig. 8), suggesting a broad involvement of *FGD3* in the orchestration of the tumor immune microenvironment.

**Fig. 9:**
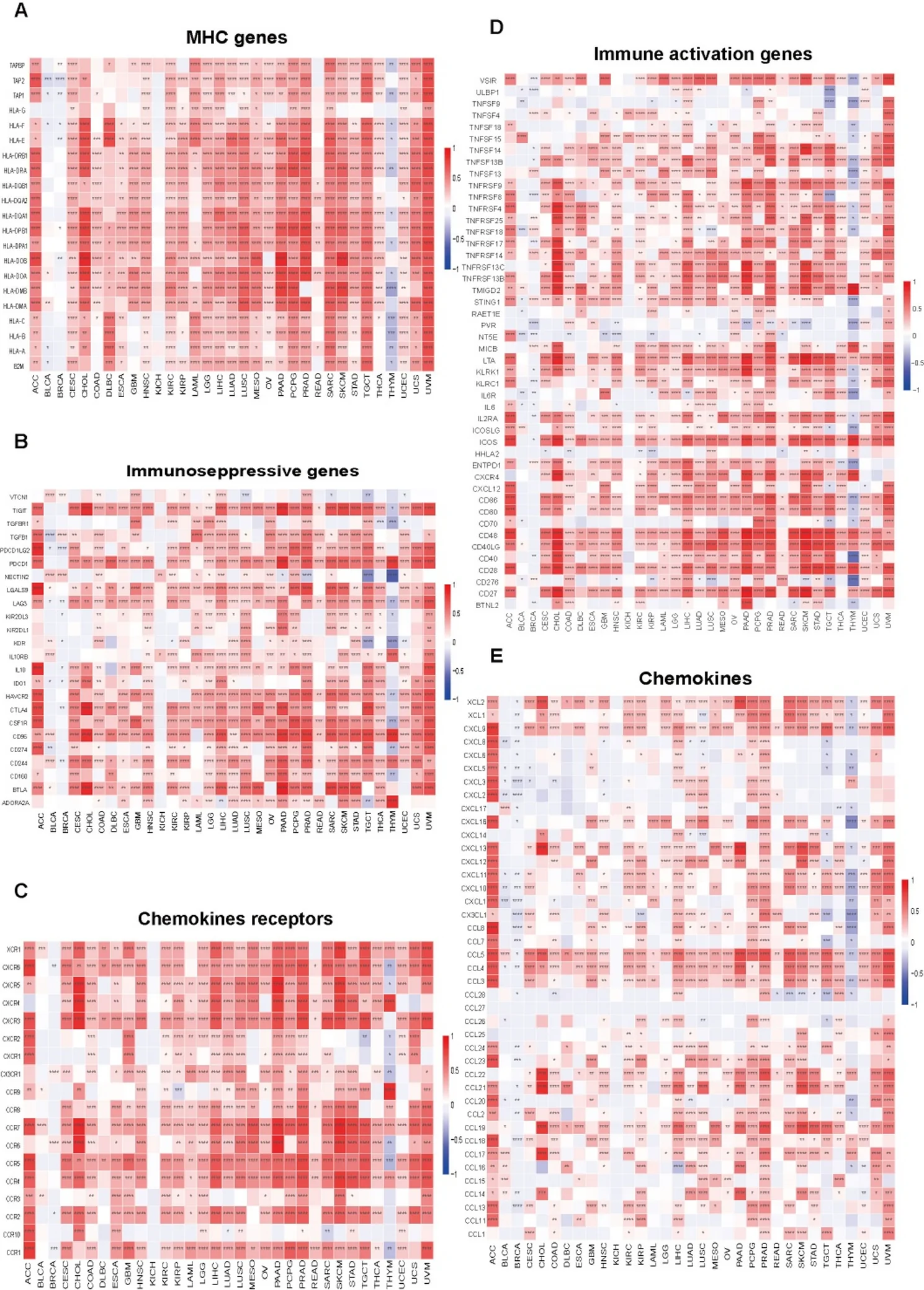
Relationship between *FGD3* expression and immune-related factors. Associations between *FGD3* expression and (**A**) chemokine receptors, (**B**) chemokines, (**C**) immunosuppressive factors, (**D**) immunostimulatory factors, and (**E**) MHC genes are shown. Statistical significance is indicated as *p < 0.05, **p < 0.01, ***p < 0.001, and ****p < 0.0001. All results correspond to FDR-adjusted p-value < 0.05 across cancer types

Further analysis revealed that, across the majority of TCGA cancer types, *FGD3* expression was positively associated with infiltration of B cells, CD4⁺ T cells, Tregs, CD8⁺ T cells and macrophages. In contrast, in specific tumor contexts, notably SARC and THYM, *FGD3* expression was negatively correlated with MDSC and progenitor cell infiltration, suggesting a context-dependent immunoregulatory role. Given the pivotal role of immune cell infiltration in tumor development and therapeutic responsiveness, the observed correlations between *FGD3* expression and immune populations indicate that *FGD3* may actively participate in tumor-immune crosstalk, potentially influencing both disease progression and response to immunotherapy (Fig. 9A-E). Collectively, these findings, at the level of immune cell composition, reinforce the intimate association of *FGD3* with tumor immunity and provide a cellular and mechanistic foundation for subsequent analyses of its immunological function and prognostic relevance.

### *FGD3* Expression Biological Significance in Tumours

To systematically elucidate the potential biological functions of *FGD3* across diverse tumor types, GSEA was employed to investigate its associated signalling pathways. Samples were stratified into high- and low-expression groups based on median *FGD3* expression within each cancer type, and GSEA and GSVA were then performed to evaluate associated biological processes.

**Fig. 10:**
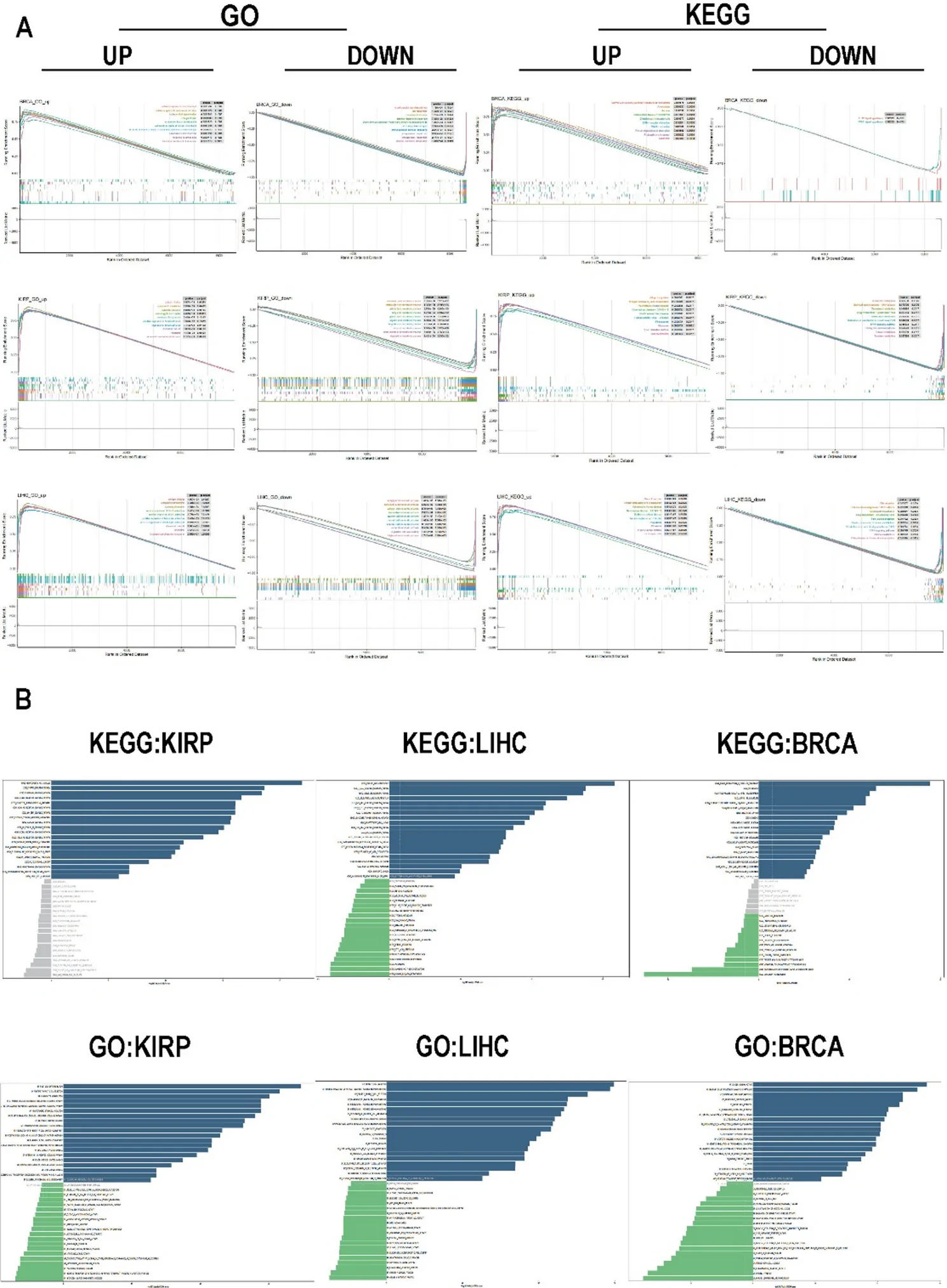
Functional analysis of *FGD3*. GSEA results of *FGD3* in BRCA, ACC, and UCEC based on GO functional annotation and KEGG pathway analysis are shown in Fig. 10A. Curves with different colors indicate enriched functions or pathways across cancer types, where enrichment score peaks represent positive regulation and troughs represent negative regulation. GSVA analysis based on GO and KEGG datasets in BRCA, ACC, and UCEC is shown in Fig. 10B. Bars indicate pathways with the most significant positive correlation (blue), most significant negative correlation (green), and no significant correlation (grey, FDR > 0.05). The horizontal axis represents -log10(p-value) of GSVA scores. Drug sensitivity correlations were adjusted for multiple testing using FDR, with significance defined as q-value < 0.05

GSEA revealed that elevated *FGD3* expression in BRCA, ACC, and UCEC was significantly associated with multiple immune-related biological processes, including the unfolded protein response, IRE1-mediated unfolded protein response, antigen processing and MHC class I presentation, immunoglobulin-mediated humoral immunity, and cellular responses to misfolded proteins. Complementary KEGG pathway analysis further indicated that *FGD3* is closely linked to antigen processing and presentation, Th17 cell differentiation, IL-17 signalling, and endoplasmic reticulum protein processing pathways, suggesting a link between *FGD3* expression and immune activation in the tumor microenvironment. Further analyses showed that high *FGD3* expression in BRCA and UCEC was positively correlated with adaptive immune responses, B-cell receptor signalling, and B-cell activation. Conversely, in BRCA, *FGD3* was negatively associated with immunoglobulin complex formation and RNA degradation processes, while in UCEC, it is negatively associated with immune response activation signalling pathways (Fig. 10A), indicating that *FGD3* may exert context-dependent bidirectional immunomodulatory effects across different tumor types.

To further validate these functional characteristics and assess pathway activity, GSVA was performed. Among the top 15 pathways associated with *FGD3* expression, multiple immune-related pathways were identified alongside tumor-related processes such as ubiquitin-mediated proteolysis, non-homologous end joining, autophagy regulation, mismatch repair, basal DNA repair, protein export, and cell cycle control (Fig. 10B). Additional associations were observed with basal transcription factors, GPI-anchor biosynthesis, and RNA splicing. Notably, *FGD3* expression displayed a negative correlation with energy metabolism-related pathways in BRCA and UCEC, whereas in ACC, it was inversely associated with immunoglobulin receptor activity and related immune pathways.

### Association of *FGD3* expression with drug sensitivity and molecular docking of *FGD3*-targeted compounds

**Fig. 11:**
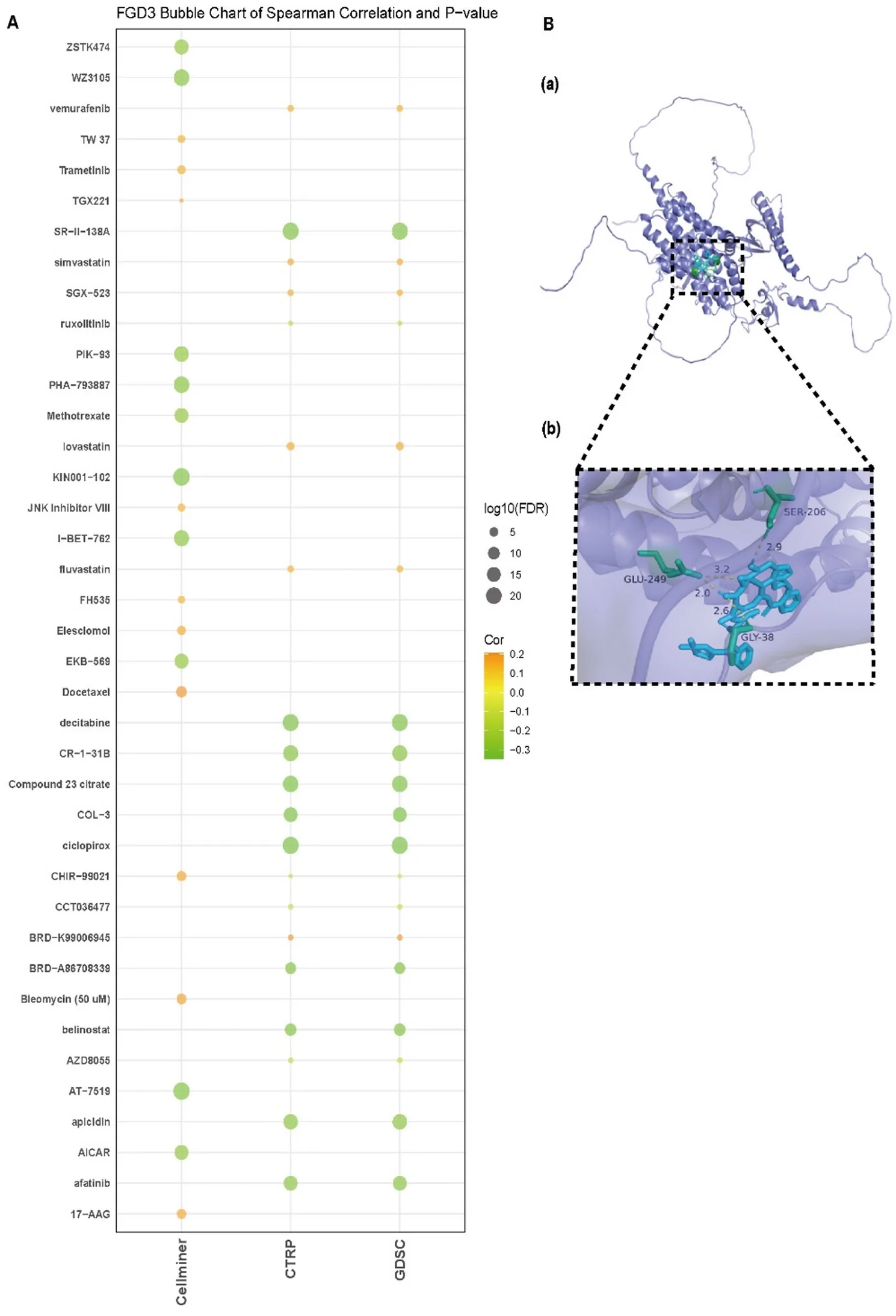
*FGD3* expression, drug sensitivity, and molecular docking of *FGD3*-targeted compounds. The relationship between *FGD3* expression and predicted drug response in a panel of NCI-60 cancer cell lines is shown in Fig. 11A. Molecular docking analysis of Docetaxel with the *FGD3* protein is shown in Fig. 11B. The protein structure representation of *FGD3* and the ligand structure of Docetaxel are shown in Fig. 11B(a). A close-up view of the interaction between Docetaxel and the *FGD3* protein is shown in Fig. 11B(b). Important receptor residues are represented as sticks, with the receptor shown in cartoon representation (purple and green), Docetaxel shown in blue, and binding residues shown in green

To assess the potential predictive value of *FGD3* in anti-cancer therapy, the correlation between *FGD3* expression and drug responses was systematically evaluated. As illustrated in Fig. 11A, *FGD3* expression exhibited significant positive correlations with the efficacy of multiple agents, including vemurafenib, TW-37, trametinib, simvastatin, lovastatin, JNK inhibitor VIII, fluvastatin, FH535, linsitinib, docetaxel, CHIR-99,021, bleomycin, AZD8055, and 17-AAG, whereas negative correlations were observed with ZSTK474, WZ3105, SR-II-138 A, PIK-93, PHA-793,887, methotrexate, KIN001-102, I-BET-762, EKB-569, desipramine, CR-1-31B, compound 23 citrate, COL-3, cyclosporone, BRD-A86708339, belinostat, AT-7519, afatinib, AICAR and alectinib. These findings suggest that *FGD3* expression may modulate tumor sensitivity to diverse classes of anti-cancer agents.

To further investigate potential molecular interactions between *FGD3* and candidate drugs, a structure-based docking analysis was performed using compounds selected from the CellMiner database. AutoDock4 was employed to predict drug-protein binding sites and affinities. Docetaxel, a first-line chemotherapeutic agent widely used in multiple malignancies including breast cancer [44, 45], was selected for in-depth analysis due to its clinical relevance and reliance on drug sensitivity. Docking results revealed that docetaxel formed stable interactions with *FGD3* through multiple hydrogen bonds and pronounced electrostatic contacts (Fig. 11B), with an optimized binding energy of -5.6 kcal/mol, indicating strong binding stability.

Taken together, *FGD3* expression not only correlates with the response to multiple anti-cancer drugs but also exhibits a structurally stable interaction with docetaxel, supporting its potential as a predictive molecular biomarker for chemotherapy sensitivity.

### Silencing *FGD3* causes cell proliferation, migration

**Fig. 12:**
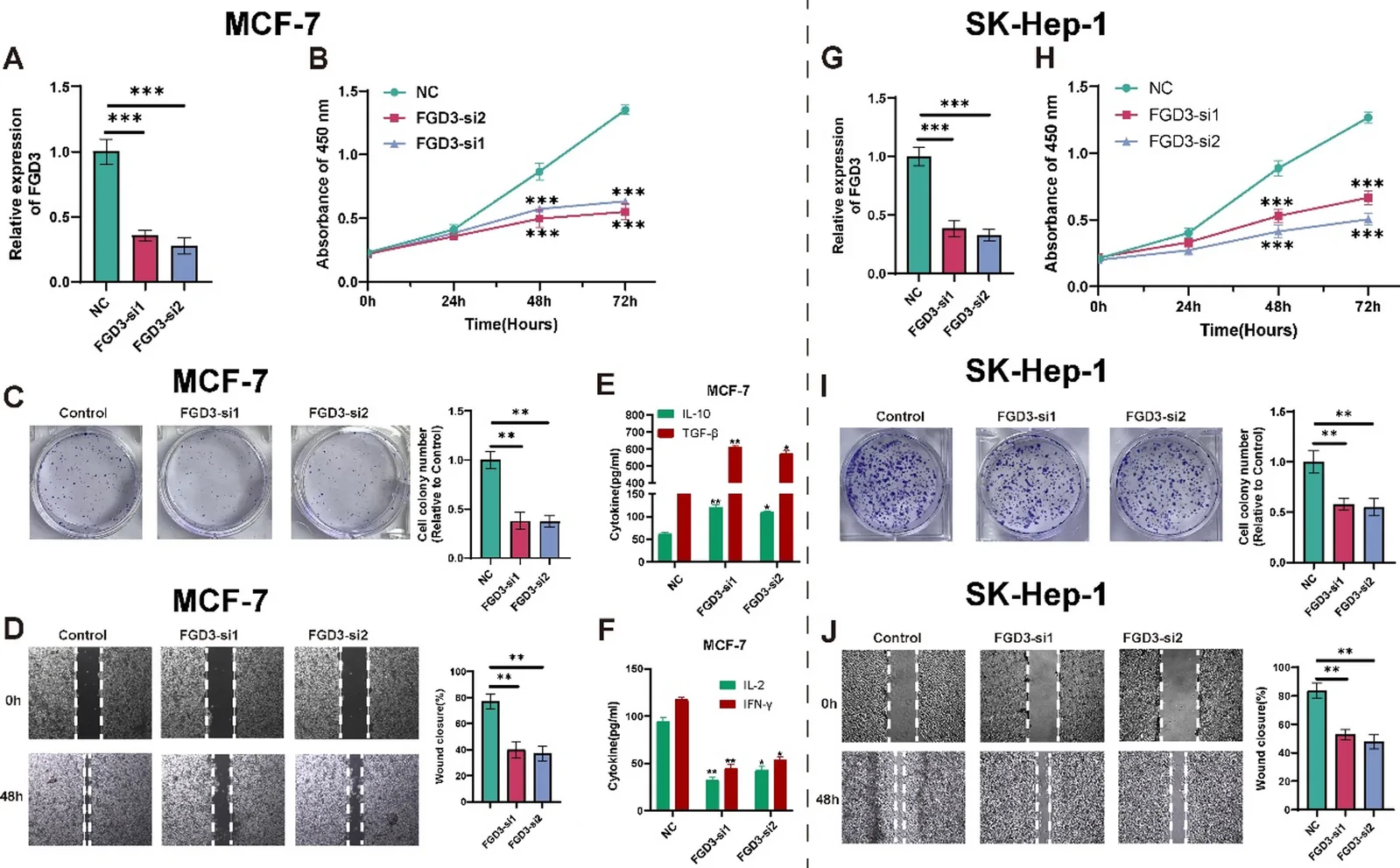
*FGD3* suppression alters cellular proliferation and migration. The relative expression levels of *FGD3* in MCF-7 breast cancer cells and SK-Hep-1 liver adenocarcinoma cells transfected with *FGD3*-si1, *FGD3*-si2, or NC are shown in Figure **A** and Figure **G**, respectively. CCK-8 assays showing the effects of *FGD3* knockdown on the growth of breast cancer cells and liver adenocarcinoma cells are presented in Figure **B** and Figure **H**. Colony formation assays comparing clonogenic potential among *FGD3*-si1, *FGD3*-si2, and NC groups are shown in Figure **C** and Figure **I**. Wound healing assays evaluating migratory capacity are shown in Figure **D** and Figure J. Significance levels are indicated as *p < 0.05 and **p < 0.01. Cytokine secretion profiles following co-culture are shown in Figure **E** and Figure **F**. Figure E shows concentrations of anti-inflammatory cytokines IL-10 and TGF-β, while Figure **F** shows pro-inflammatory cytokines IL-2 and IFN-γ. All experiments were conducted using hPBMCs derived from at least three independent donors. Data are presented as mean ± SD. Statistical significance was assessed using one-way ANOVA followed by Tukey’s post hoc test

The relationship between *FGD3* expression and cancer cell proliferation was investigated through siRNA-mediated silencing of *FGD3*. Breast cancer and liver adenocarcinoma cell lines were categorized into three groups: NC, *FGD3*-si1 and *FGD3*-si2, to assess the functional consequences of *FGD3* depletion. Experimental results demonstrated a pronounced reduction in proliferative capacity concomitant with decreasing *FGD3* levels. Specifically, across the three experimental groups, *FGD3* expression declined progressively over time, as indicated by optical density measurements (Fig. 12A, B, G, H). This observation suggests a correlation between *FGD3* expression and the proliferative state of cancer cells.

Furthermore, *FGD3* silencing markedly attenuated the proliferation of breast cancer cells and liver adenocarcinoma cells. Cells with reduced *FGD3* expression exhibited diminished growth during culture, highlighting the pivotal role of *FGD3* in sustaining tumor cell proliferation (Fig. 12C, I). The impact of *FGD3* downregulation on cancer cell motility was also assessed. Silencing of *FGD3* led to a significant impairment in migratory capacity (Fig. 12D, J), indicating that elevated *FGD3* expression not only promotes proliferation but may also contribute to enhanced migratory capacity, which is an important feature associated with tumor progression.

In addition, cytokine analysis revealed that *FGD3* knockdown significantly altered the inflammatory cytokine profile in MCF-7 cells. The secretion of the anti-inflammatory cytokines IL-10 and TGF-β was significantly increased, whereas the levels of the pro-inflammatory cytokines IL-2 and IFN-γ were markedly decreased following *FGD3* silencing (Fig. 12E, F). These findings suggest that *FGD3* may be involved in regulating the inflammatory state of tumor cells.

Collectively, these findings indicate that *FGD3* expression is associated with the proliferative and migratory capacities of cancer cells, suggesting a potential role in tumor progression and regulation of the tumor microenvironment.

## Discussion

Cancer immunotherapy, particularly immune checkpoint blockade (ICB), has emerged as a transformative strategy in oncology. However, its clinical efficacy remains highly variable, largely due to the intrinsic heterogeneity of the TME, which critically shapes antitumor immune responses and therapeutic outcomes [46]. Although biomarkers such as PD-L1 expression and tumor mutational burden have been widely investigated, their predictive value remains inconsistent across tumor types and patient populations. These limitations highlight the urgent need for more robust biomarkers that better capture TME complexity.

*FGD3* is primarily localized in the cytoplasm, where its interaction with small GTPases plays a key role in regulating cytoskeletal dynamics and cellular processes. Previous large-scale studies have consistently suggested a protective role of *FGD3* in breast cancer. Notably, analyses from the Sage Bionetworks-DREAM Breast Cancer Prognosis Challenge first identified *FGD3* as a gene strongly associated with favorable outcomes [47]. Subsequent studies further confirmed that reduced *FGD3* expression correlates with aggressive tumor features, including increased lymph node metastasis and poor survival [12, 24]. Importantly, these associations appear to be independent of established clinical variables such as ER status and molecular subtype, supporting its potential as a robust prognostic biomarker.

Survival analyses across OS, DSS, DFI, and PFS demonstrated a significant association between *FGD3* expression and patient prognosis. Notably, the direction of this association was highly context-dependent across cancer types, with high *FGD3* expression associated with poor outcomes in LGG and UCS, but favorable prognosis in LIHC and SARC. This bidirectional pattern suggests that *FGD3* does not function as a universal prognostic biomarker but instead exhibits tumor-type-specific biological effects that are likely shaped by the molecular and immune context of each malignancy. These findings further indicate that the prognostic value of *FGD3* should be interpreted within a precision oncology framework, in combination with genomic and immunological features such as TMB, MSI, and immune infiltration patterns. Such an integrative approach may provide a more comprehensive understanding of survival variability across malignancies.

Analysis of *FGD3* genomic alterations using cBioPortal revealed that copy number variations (CNVs) were particularly prevalent in UCEC. Further investigation demonstrated that CNVs may contribute to dysregulated *FGD3* expression, suggesting a potential functional link between genomic instability and transcriptional regulation. In ACC, *FGD3* expression showed positive correlations with both CNV burden and DNA methylation levels, indicating that genetic and epigenetic mechanisms may jointly regulate its activity. These findings are consistent with the broader concept that CNVs and DNA methylation are key determinants of gene expression variability in cancer and may contribute to tumor-specific phenotypes [48, 49].

TMB has been recognized as a broadly applicable prognostic feature post-ICI therapy [50] and can guide regimen selection in the precision oncology era [51]. Similarly, MSI and its association with features such as elevated TMB and lymphocyte infiltration are established predictors for PD-1 inhibitor response [52]. Our analyses revealed that *FGD3* expression correlates with TMB in eight cancer types and with MMR in seven, suggesting that *FGD3* may reflect genomic instability and DNA repair status, factors that collectively influence ICI efficacy. Moreover, correlation analyses in independent cohorts indicate that *FGD3* expression may serve as a predictive biomarker for ICI sensitivity, including anti-PD-1 therapy in melanoma. The positive association between *FGD3* and MMR gene expression across multiple tumors, including CESC and UVM, further supports a potential mechanistic link whereby *FGD3* modulates the TME and DNA repair pathways. These findings underscore the potential utility of integrating *FGD3* expression into immunotherapy stratification frameworks to optimize patient selection and enhance therapeutic outcomes.

Our data suggest a potential role for *FGD3* in modulating the TME. The composition and characteristics of the TME not only influence tumor response to ICIs but also correlate with clinical outcomes [53]. ESTIMATE analyses revealed a negative correlation between *FGD3* expression and stromal, immune, and ESTIMATE scores across 33 cancer types, indicating that *FGD3* may contribute to a suppressive TME and reduced immune activity, potentially facilitating tumor progression [54]. *FGD3* expression was also associated with infiltration of multiple immune cell types, including B cells, CAFs, lymphoid progenitors, CD8 + T cells, and neutrophils, highlighting a link between *FGD3* and TME cellular composition. In KIRC, elevated *FGD3* expression correlated with M2-type macrophage infiltration, consistent with worse prognosis, suggesting that immune cell interactions may partly mediate *FGD3*’s prognostic effect. Pathway analyses further implicated *FGD3* in immune regulation via cytokine-related signaling, including Th17 and IL-17 pathways, as well as potential roles in MHC-I-dependent cytotoxic T-cell activation. Although these findings are correlative, they provide a rationale for future functional studies, such as cytokine profiling, to clarify the direct immunomodulatory mechanisms of *FGD3*.

These findings provide additional insights into *FGD3*’s role in oncology, particularly its association with immune cells and immune-related genes. Across most cancer types, *FGD3* expression positively correlates with genes involved in MHC, immune activation and suppression, chemokines, and chemokine receptors, suggesting that *FGD3* may influence prognosis through modulation of immune infiltration. Analyses using cBioPortal indicated that *FGD3* alterations are more frequent in BRCA, while in ACC, *FGD3* mutations showed the strongest correlation with survival outcomes. Consistently, high *FGD3* expression in BRCA was associated with better prognosis, as supported by HPA data, reinforcing its potential utility as a context-specific prognostic marker.

GSEA analyses indicated that high *FGD3* expression is associated with immune regulation and stress response processes, including ubiquitin-mediated proteolysis, NHEJ, MMR, BER, protein export, and cell cycle pathways. These pathways are critical for DNA repair, stress adaptation, and cell cycle control, suggesting that *FGD3* may shape the TME through multiple cellular mechanisms. GSVA further confirmed that *FGD3* correlates with immune- and immune factor-related pathways across diverse cancer types, highlighting its potential role as an immunomodulatory factor. From a translational perspective, the impact of *FGD3* appears context-dependent. In tumors with low *FGD3* expression, such as BRCA, restoring *FGD3* activity might suppress tumor progression, whereas in cancers with high *FGD3* expression within an immunosuppressive TME, therapeutic strategies could benefit from combination with ICIs. These observations emphasize that interventions targeting *FGD3* should consider tumor-specific biology and immune context to optimize efficacy.

Subsequent analyses explored potential anticancer drugs that may exert effects by modulating *FGD3*. Drug sensitivity profiling suggested that docetaxel, a widely used taxane for BRCA, lymphoma, lung, and ovarian cancers [55], could interact with *FGD3*-related pathways. Its known mechanisms include microtubule stabilization, inhibition of DNA replication and transcription, and induction of apoptosis, as well as modulation of immune cells such as promoting differentiation toward M1 macrophages [44, 55, 56]. These findings raise the possibility that docetaxel’s efficacy may be influenced by *FGD3* activity, a hypothesis that warrants further functional validation. More broadly, targeting *FGD3* may provide a context-dependent strategy for developing molecular therapies, particularly in BRCA and other *FGD3*-associated malignancies.

Functional validation in MCF-7 cells and SK-Hep-1 cells demonstrated that *FGD3* knockdown promotes proliferation and migration, supporting its potential role in suppressing BRCA progression. While our analyses integrate multiple databases, limitations remain, particularly methodological biases inherent to transcriptomic data. Further in vitro and in vivo studies are warranted to confirm these findings and to clarify the mechanistic basis of *FGD3*’s tumor-suppressive effects, thereby enhancing the translational relevance of this work.

Although our pan-cancer analyses reveal widespread associations between *FGD3* and the immune landscape, and in vitro experiments suggest functional involvement, these findings remain largely correlative. The precise mechanisms by which *FGD3* modulates immune cell recruitment and function require further investigation using advanced experimental models, such as comprehensive immune co-cultures and in vivo studies. Additionally, as this pan-cancer study was exploratory, prognostic associations were assessed without full adjustment for clinical confounders, including tumor stage and molecular subtype. Future work should incorporate multivariate Cox analyses and validation in independent cohorts with detailed clinical annotation to establish the independent prognostic value of *FGD3*.

In summary, *FGD3* appears to play a significant role across multiple cancer types, with expression levels associated with immune regulatory factors, immune cell infiltration, TME, TMB, and MSI, albeit with context-dependent effects. Beyond its role in normal transcriptional regulation, *FGD3* may influence tumor initiation, progression, and immune interactions. Further studies are warranted to elucidate *FGD3*-related pathways and their mechanistic contributions, which could inform future strategies for cancer diagnosis, prognosis, and therapy.

## Supplementary Information

Supplementary Material 1

## Data Availability

No datasets were generated or analysed during the current study.
